# Study on pore structure and the mechanical properties of sandstone-concrete binary under freeze–thaw environment

**DOI:** 10.1038/s41598-023-45576-4

**Published:** 2023-10-25

**Authors:** Anlong Hu, Guobin Xue, Zhipeng Shang, Zhe Cao, Xiaoping Wang, Yintao Fu, Xiaoqing Huang

**Affiliations:** 1 Economic and Technological Research Institute of State Grid Gansu Electric Power Company, Lanzhou, 730050 Gansu China; 2 Key Laboratory of Geological Hazards On Three Reservoir Area, Ministry of Education, China Three Gorges University, Yichang, 443002 Hubei China

**Keywords:** Civil engineering, Natural hazards

## Abstract

In China's cold region water conservancy and hydropower projects, the contact interface between the dam and the reservoir bank rock is prone to cracking under external loading and freeze–thaw action, which may lead to dam-bank failure and damage and cause engineering disasters. The *NMR* (Nuclear Magnetic Resonance) tests and uniaxial compression tests of concrete, sandstone, and sandstone-concrete composite after different numbers of freeze–thaw cycles were carried out to analyze the pore structure development and uniaxial compression mechanical properties of the three types of specimens under different numbers of freeze–thaw cycles. The results show that freeze–thaw cycling promotes the development of pores in sandstone and concrete, and sandstone is more sensitive to low-temperature freeze–thaw than concrete. The *UCS* (uniaxial compressive strength) of the sandstone-concrete binary changed in a *V*-shaped with the increase of the dip angle of the cemented interface, and the angle had no obvious effect on the microscopic pores. The freeze–thaw effect on the deterioration of the microscopic pore structure and mechanical properties of the sandstone-concrete binary has a similar effect pattern, in which the deterioration rate of porosity and compressive strength is faster in the early freeze–thaw period, slower in the middle period, and increases in the later period compared with the middle period, but the increase is smaller than that in the early period of freeze–thaw. In addition, the relationship between the porosity and *UCS* of the sandstone-concrete binary under the freeze–thaw cycle environment is a quadratic parabola.

## Introduction

In recent years, China has accelerated the construction of a strategic system of clean, efficient, safe and sustainable development, especially in the western region, as an important support for the national energy, and has successively developed and constructed a large number of water conservancy and hydropower projects, mining and energy extraction and underground energy storage projects involving large-scale rock engineering. While the geological structure of the western region is complex, and there are many cold areas. Repeated freezing and thawing lead to changes in the physical properties of the rock and soil, thus affecting the stability of the project^1–4^. Therefore, the study of freeze–thaw on the microstructure and mechanical behavior of rock-supported concrete can provide a theoretical basis for the safe construction and operation of engineering in cold areas.

At present, scholars at home and abroad have conducted in-depth studies on the mechanical behavior of freeze–thaw rocks^5–8^. Zhu et al.^9^ proposed a numerical calculation method based on granular flow expansion and simulated the deformation and rupture evolution of sandstone under the coupled stress-freezing–thawing action using the freeze-thawing deformation law of sandstone obtained from indoor tests. Jiang et al.^10^ conducted uniaxial compression tests on red sandstone under different freeze–thaw cycles and established the relationship between mechanical parameters and the number of freeze–thaw cycles. Shen et al.^11,12^ prepared sandstone-concrete composite material, conducted freeze–thaw cycle and *NMR* (nuclear magnetic resonance) tests and studied the pore change rule and micro-stripping process of the sandstone-concrete interface. For rock-concrete binary materials, current research has focused on shear mechanical properties and interfacial contact properties, and a few researchers have studied the compressive strength and other properties of rock-concrete composite specimens^13,14^. Selcuk et al.^15^ investigated the strength and damage behavior of sandstone-concrete composite specimens by uniaxial compression tests and the influence of the interface tilt angle on the strength and damage of the composite layer and the influence law of the interface tilt angle on the strength and damage pattern of the composite layer. Huang et al.^16^ investigated the fatigue damage and energy evolution process of rock-concrete composite specimens under cyclic loading. Vladimir et al.^17^ analyzed the interfacial shear characteristics of concrete-rock mass, established the functional analytic form of shear deformation and shear strength of rock mass with normal direction and shear load, and deduced the mathematical model which can be used for structural analysis in the preliminary design stage of a concrete dam. Yang et al.^18^ conducted compression tests of rock-fiber concrete composite structures with the help of 3D-DIC full-field strain technology and analyzed the deformation characteristics, damage characteristics, and failure modes of composite layers. In order to study the damage evolution of shotcrete lining under the action of the freeze–thaw cycle in cold area tunnels, Zhang et al.^19^ proposed an improved general state weekly dynamics (OSBPD) model by introducing the freeze–thaw damage function, considering the difference of mechanical behavior of shotcrete under tensile and compressive action. In terms of freeze–thaw microstructure, Xue et al.^20^ investigated the influence of service load and dry and wet freeze–thaw environment on the initial damage of concrete, and found that with the increase of dry and wet freeze–thaw cycles, micro and small holes in concrete evolved into medium and large holes. Liu et al.^21^ conducted several freeze–thaw cycle tests on sandstone samples and revealed the evolution law of microscopic damage during the freeze–thaw cycle using *NMR*.

In summary, there are fewer existing studies on the microscopic pore structure and mechanical behavior of rock-concrete binary materials under freeze–thaw environments, which are prevalent in the engineering community. Studying microscopic pore structure and mechanical properties of rock-concrete binary materials under a freeze–thaw environment has important practical significance for engineering design, safe construction, and operation in cold regions. In view of this, this study quantifies and evaluates the effects of freeze–thaw cycles on the pore structure and mechanical properties of rock-concrete composite specimens by making rock-concrete binary specimens with different inclination cementation interfaces indoors from the viewpoint of the cementation contact surfaces of hydroelectric dams and dams and reservoir banks. Furthermore, freeze–thaw cycles, *NMR* and mechanical properties tests were conducted using *T2* spectra, porosity, and compressive strength.

## Experimental materials and scheme

### Specimen preparation

**Figure 1:**
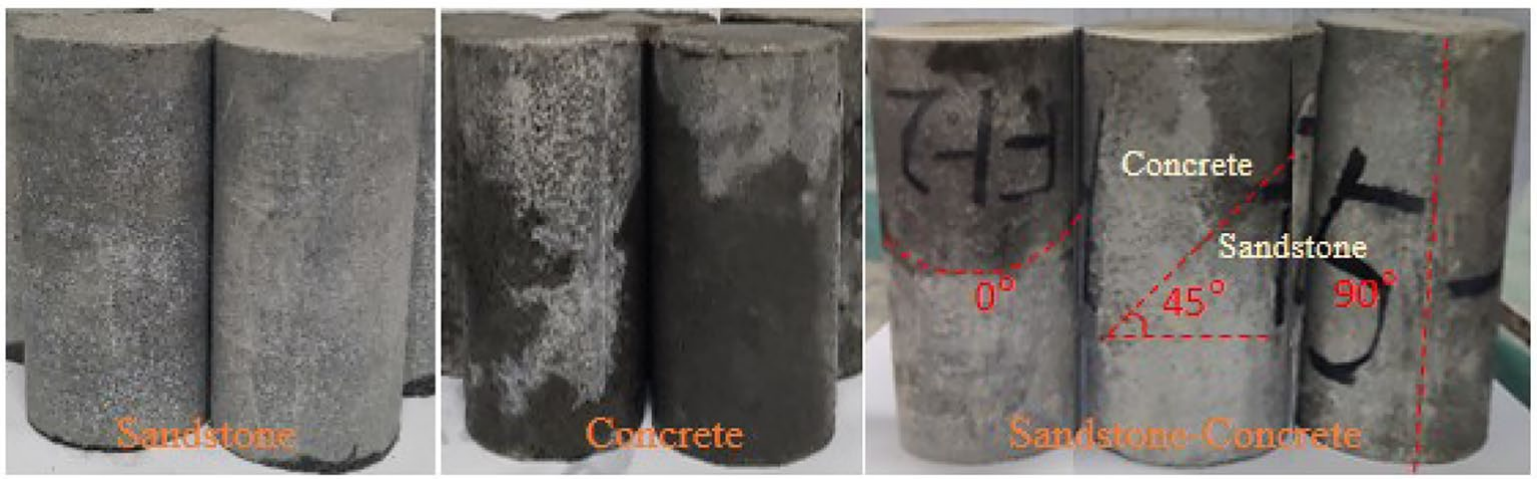
Types of prepared specimens.

The sandstone used for the test was selected from Sichuan Province, China, which is grayish-white in color, coarse-grained in structure, with moderate porosity and uniform distribution. The collected sandstone was processed into a standard cylindrical specimen of 50 mm × 100 mm (diameter × height) according to the International Society of Rock Mechanics (ISRM) test protocol. Rock-concrete binary composite specimens were made from C30 grade concrete with sandstone specimens of different inclination angles. The test was designed to produce sandstone, concrete, and sandstone-concrete binary body with 0°, 45°, and 90° dip angles. Five types of specimens and 42 samples were prepared. The specimens with similar ultrasonic wave velocities were then selected by manually eliminating the appearance defects (Fig. 1).

### Experimental program

**Figure 2:**
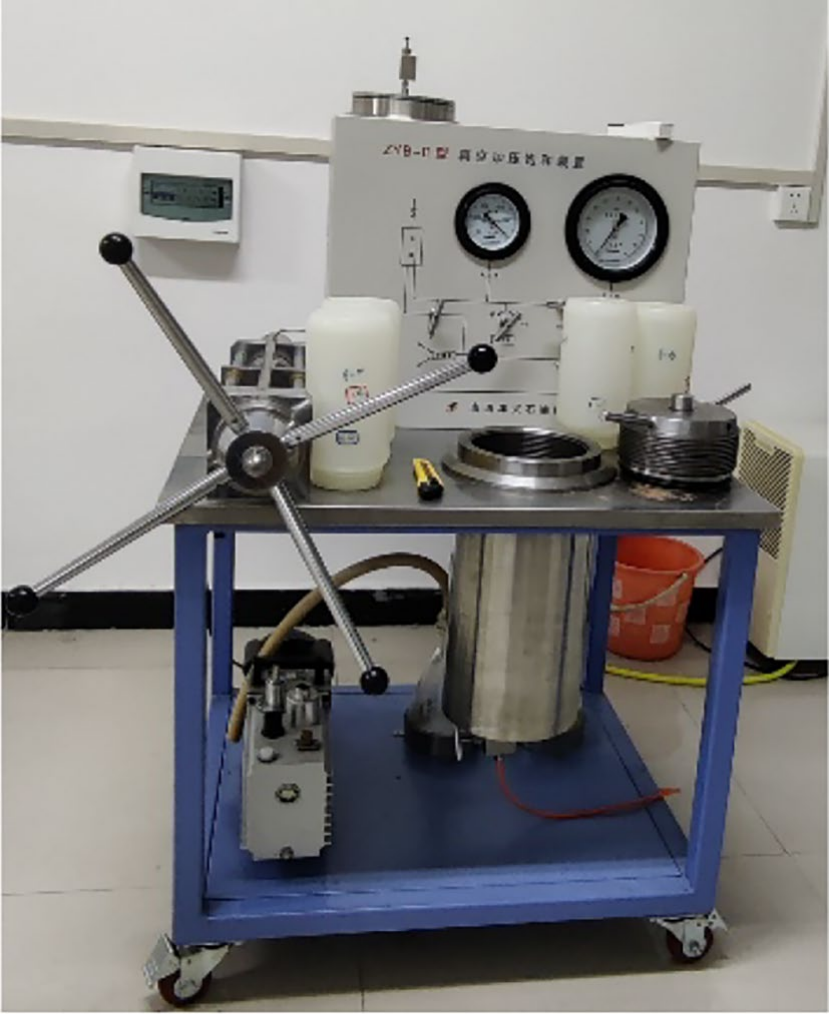
Vacuum saturator.

**Figure 3:**
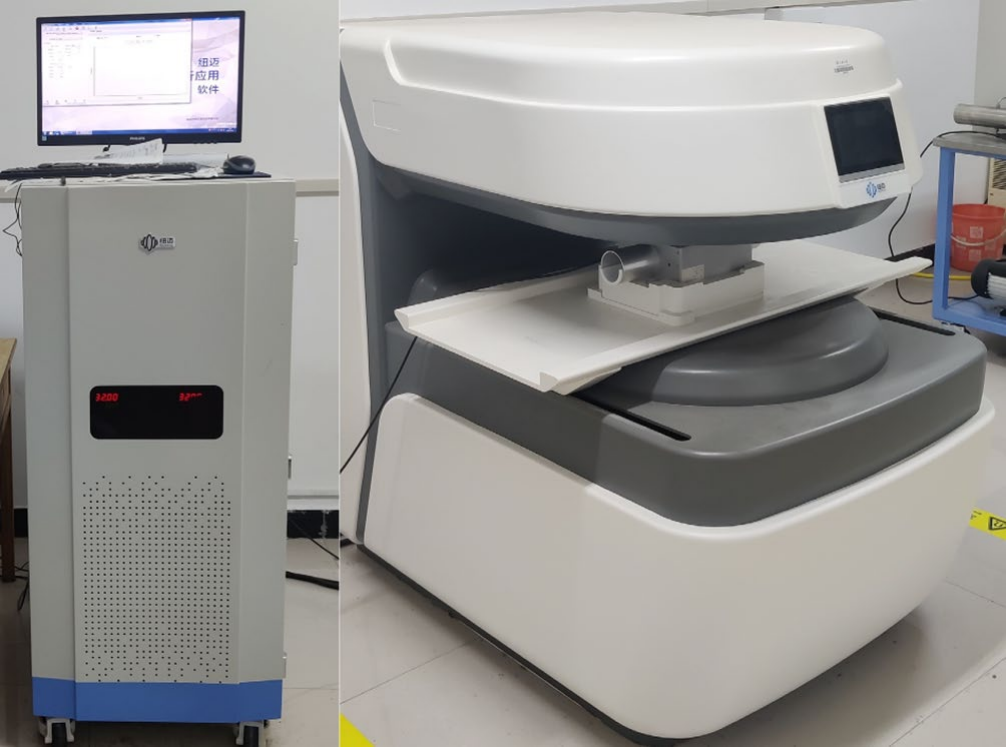
The *MacroMR12-150H-I NMR* analysis system.

**Figure 4:**
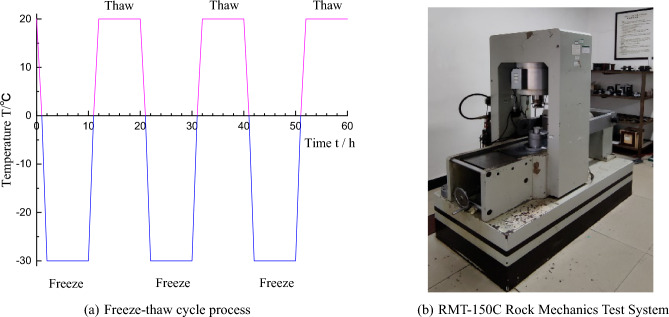
Test procedure and mechanical testing equipment.

1. Water saturation and *NMR* test. The specimens were immersed in distilled water for 24 h and then put into a vacuum water saturation machine (Fig. 2) with a vacuum pressure of 100 kPa for 48 h. Then, the specimens were put into the water tank for 24 h. The *MacroMR12-150H-I NMR* analysis system, as shown in Fig. 3, was used to saturate the specimens. The specimens were scanned, and the relevant parameters were measured.
2. Freeze–Thaw cycle process. The temperature change curve of the freeze–thaw cycle process is shown in Fig. 4a. The number of freeze–thaw cycles was designed to be 0, 20, 40 and 60 cycles, with freezing for 10 h and thawing for 10 h. The whole freeze–thaw cycle of 20 h was one cycle.
3. Mechanical properties test. *RTR-1500* testing machine, as shown in Fig. 4b, was used to conduct a uniaxial compression test on the specimens after freeze–thaw cycles, with a loading rate of 0.1 MPa/s until the specimens were damaged, and the test was stopped.

## Experimental results and analysis

### ***T***_***2***_ spectrum characteristic analysis of NMR

**Figure 5:**
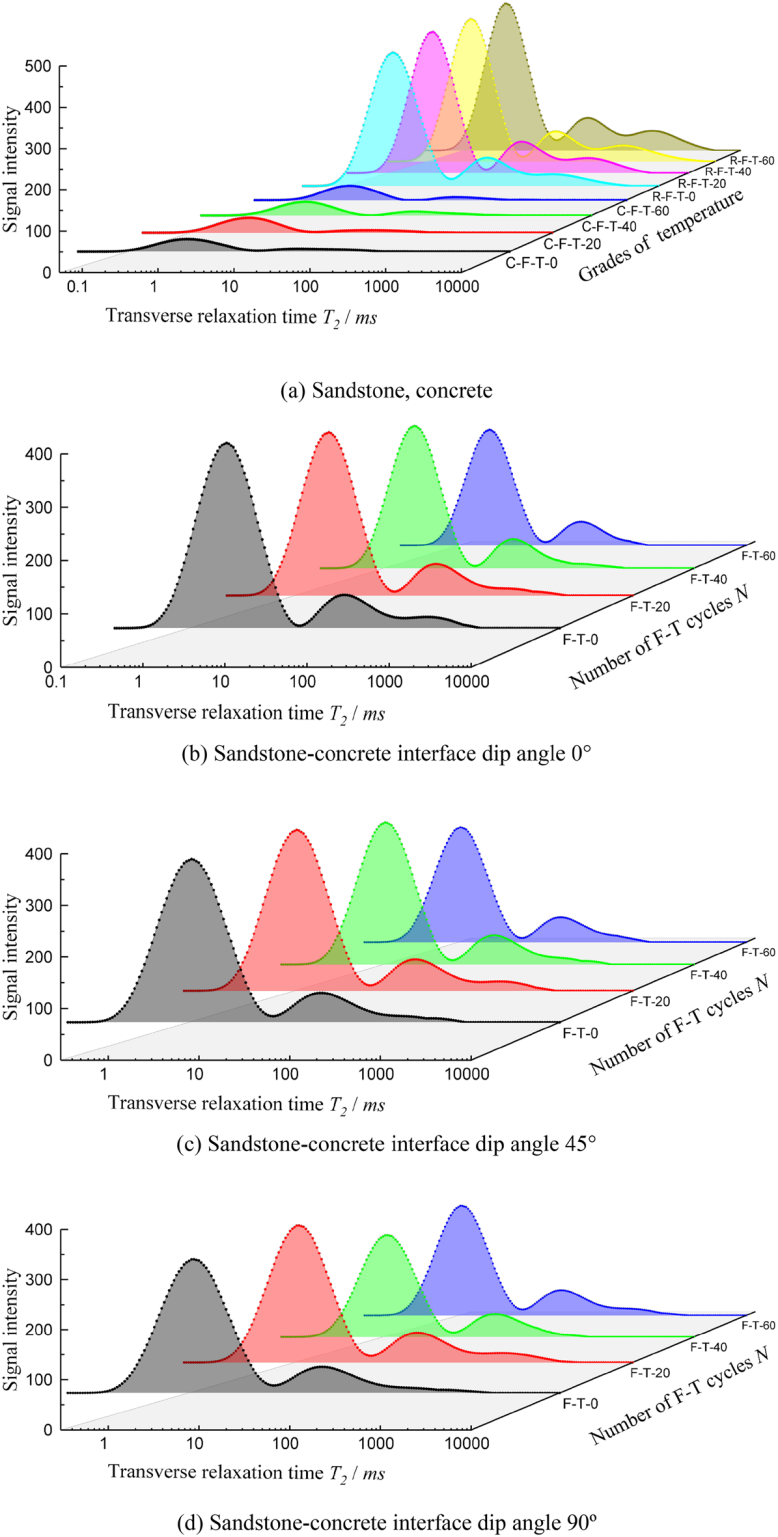
*T*_2_ spectrum curve of *NMR*.

According to the *NMR* principle, the transverse relaxation time of hydrogen-containing fluids in saturated rock specimens is proportional to the pore size^22–24^. Therefore, the *T*_*2*_ spectrum distribution can reflect the pore structure inside the rock. The obtained *T*_2_ spectral curves of the specimens in their original state and after freeze–thaw cycles are shown in Fig. 5.

Figure 5 shows that the *NMR* signal intensity amplitude of the sandstone specimen is much larger than that of the concrete specimen, and the signal intensity amplitude of the sandstone-concrete composite specimen is between the two materials. There is no obvious pattern in the effect of the angle of the cemented interface or the contact area of the cemented surface on the signal intensity amplitude of the specimen.

The *T*_2_ spectral curve shows that two spectral peaks appear in the concrete specimen, and the signal intensity amplitude of the first spectral peak is significantly larger than that of the second spectral peak, indicating that the concrete specimen is dominated by small-sized pores. The sandstone specimen and the sandstone-concrete composite specimen both showed three spectral peaks, and the 1st spectral peak was significantly larger than the 2nd and 3rd spectral peaks, but the 3rd spectral peak was less obvious. The results indicate that the sandstone specimen and the sandstone-concrete composite specimen are dominated by small-sized pores, with a small amount of medium-sized and large-sized pores. After freeze–thaw cycles, the temperature difference did not significantly change the morphology of the *T*_2_ spectral curves of various specimens, but the *NMR* signal intensity amplitude of the spectral peaks increased with the increase of the freeze–thaw times, indicating that both small pores and medium and large pores were developed with the increase of the freeze–thaw times. Furthermore, the troughs between the spectral peaks also gradually flattened out as the freeze–thaw cycle continued, i.e., the small pores of the specimens gradually developed into the medium pores, and the medium pores developed into the large pores. The results indicate that the pores of the specimens can develop to a higher degree as the freeze–thaw cycle continues.

### Analysis of specimen porosity results

According to the *NMR* measurement results, the *T*_2_ spectral area, which can reflect porosity, was calculated. The results are shown in Tables 1 and 2. The change of *T*_2_ spectral area can be analyzed to obtain the change law of the pore structure of the specimen, and then the damage degree of the specimen under the freeze–thaw cycle can be expressed quantitatively.

**Table 1:** *T*_2_ spectral area of sandstone and concrete specimens during freeze–thaw cycle.

Category	Sandstone				Concrete			
	0 Cycles	20 Cycles	40 Cycles	60 Cycles	0 Cycles	20 Cycles	40 Cycles	60 Cycles
*T*_2_ area/10^3^	18.94	20.93	22.32	24.41	3.99	4.12	4.23	4.35

**Table 2:** *T*_2_ spectral area of sandstone -concrete composite specimen during freeze–thaw cycle.

Category	Sandstone-concrete dip angle 0°				Sandstone-concrete dip angle 45°				Sandstone-concrete dip angle 90°			
	0 Cycles	20 Cycles	40 Cycles	60 Cycles	0 Cycles	20 Cycles	40 Cycles	60 Cycles	0 Cycles	20 Cycles	40 Cycles	60 Cycles
*T*_2_ area/10^3^	11.57	12.69	13.64	14.73	13.89	14.97	15.08	15.40	11.49	12.03	12.72	13.50

Tables 1 and 2 show that with the increase in the number of freeze–thaw cycles, the *T*_2_ spectral area of various specimens increases accordingly, but there are differences in the magnitude of their respective increases. The *T*_2_ spectral area of sandstone specimens increased by 28.92% after 60 freeze–thaw cycles, while that of concrete specimens increased only by 8.71%, indicating that the sandstone is more sensitive to temperature freeze–thaw cycles than concrete. The *T*_2_ spectral area of the sandstone-concrete composite specimens was affected by the joint influence of sandstone and concrete materials, and their porosity was all between the sandstone and concrete specimens. The *T*_2_ spectral area of the sandstone-concrete specimens with 0° interface inclination increased by 8%, 17.89%, and 27.35% after 20, 40, and 60 freeze–thaw cycles, respectively. After 60 freezing–thawing cycles, the maximum *T*_2_ spectral area is 15.40 × 10^–3^ and 13.50 × 10^–3^, and the maximum increase is 10.87% and 17.49%, respectively. Therefore, compared with the interface inclination of 45° and 90°, the *T*_2_ spectral area increases the most after the interface inclination of 0° goes through the freeze–thaw cycle. As can be seen from the *T*_2_ spectral area of the sample undergoing 0 freezing-thaw cycle, the *T*_2_ spectral area of the sample with an interface inclination of 45° is the maximum (13.89 × 10^–3^), the interface inclination of 0° is the middle, and the interface inclination of 90° is the minimum (11.49 × 10^–3^). There are too few test results from specimens with different interfacial inclinations to distinguish whether this law exists in itself or is caused by the dispersion of specimens. Hence, there is no obvious pattern in the effect of cementation interface inclination angle on the change of *T*_2_ spectral area, but there is an obvious pattern in the effect of the number of freeze–thaw cycles on the change of *T*_2_ spectral area.

The growth rates of the *T*_2_ spectral area of sandstone specimens and sandstone-concrete composite specimens were significantly higher than those of 20–40 and 40–60 freeze–thaw cycles during 0–20 freeze–thaw cycles, while the growth rate of the *T*_2_ spectral area of concrete was the smallest for the whole cycle. The growth rate of the *T*_2_ spectral area of sandstone-concrete composite specimens slowed down during 20–40 freeze–thaw cycles compared with 0–20 cycles, but the *T*_2_ spectral area of 40–60 cycles showed a circular growth trend. The result indicates that concrete specimens have better freezing resistance than sandstone specimens and sandstone-concrete composite specimens, and the low temperature at the beginning of freeze–thaw can cause irreversible damage to sandstone specimens and sandstone-concrete composite specimens. The damage continues to occur as the freeze-thawing continues, and when the number of cycles exceeds a certain threshold, the porosity develops again and the damage increases.

**Figure 6:**
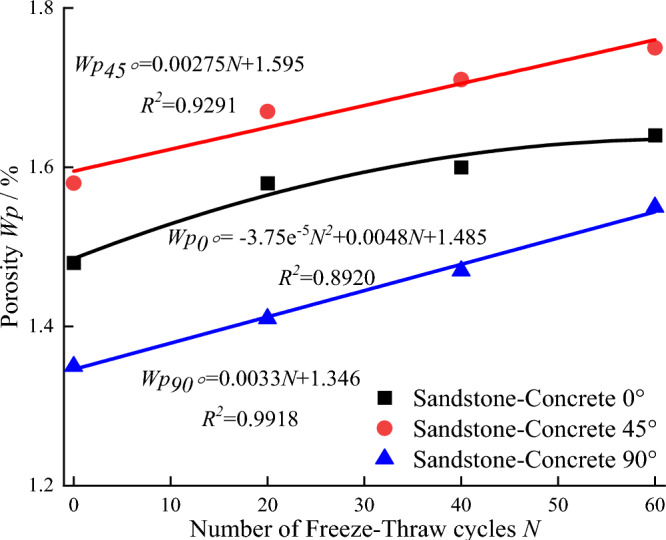
Relationship between porosity and freeze–thaw times.

Figure 6 shows the relationship between the number of freeze–thaw cycles and porosity at different inclination angles. As shown in Fig. 6, under the same freeze–thaw cycles, the porosity of the sample with an interface inclination of 90° is the smallest, the inclination of 0° is the middle, and the porosity of the sample with an Angle of 45° is the largest. The reason is that when sandstone and concrete are made into composite samples, the different area of cementing interface and the contact surface angle leads to the inconsistent infiltration of cement grout in concrete into sandstone. According to the curve fitting of the relationship between the number of freeze–thaw cycles and the porosity, the porosity of the sample with an inclination of 45° and 90° has a linear equation with one unknown relationship with the number of freeze–thaw cycles, while that of the sample with an inclination of 0° has a linear equation in two unknowns relationship with the number of freeze–thaw cycles. According to the above analysis, the porosity of the samples with three inclination angles increases with the increase of freeze–thaw cycles, indicating that the freeze–thaw effect promotes the development of pores inside the samples.

## Analysis of mechanical properties of sandstone-concrete dual structure after a freeze–thaw cycle

### Mechanical properties of sandstone-concrete binary

**Figure 7:**
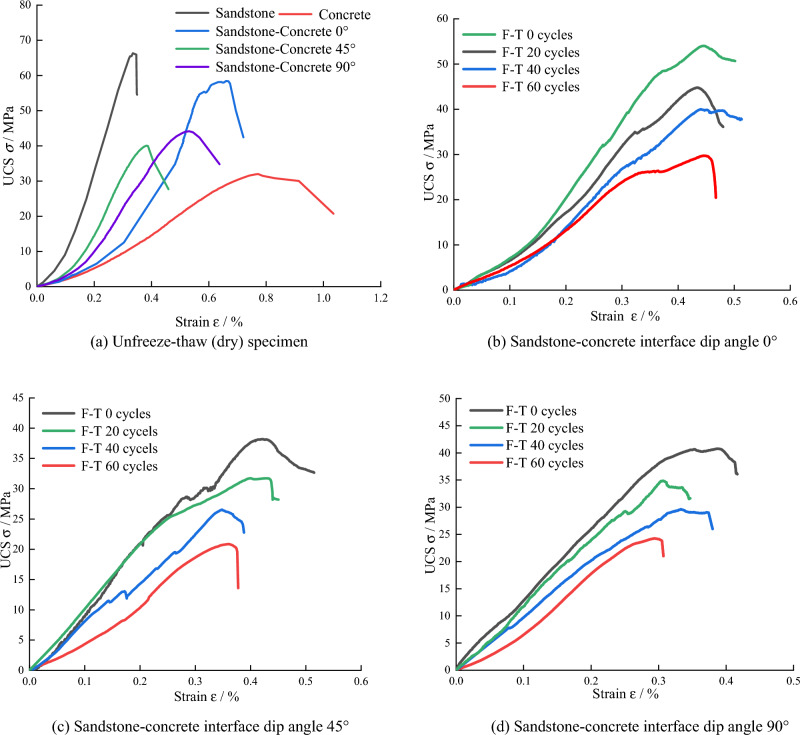
Stress–strain curves of specimens under uniaxial compression.

**Figure 8:**
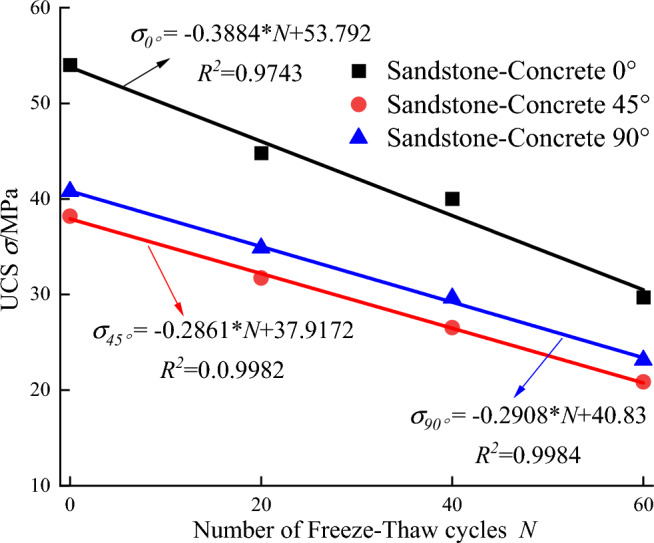
Relation between *UCS* of specimen and freeze–thaw frequency.

The uniaxial compressive stress–strain curves and the variation of strength and porosity with the number of freeze–thaw cycles of various specimens after freeze–thaw are shown in Figs. 7 and 8, respectively.

Figure 7 shows that the *UCS* (uniaxial compressive strength) of sandstone-concrete composite specimens in the dry state is between the strength of sandstone and concrete and changes with the change of the dip angle of the cemented interface. The *UCS* of the sandstone-concrete composite specimens in the dry state or after freeze-thawing in the water-saturated state showed a *V*-shaped variation pattern at the three angles of 0°, 45°, and 90° of the cementation interface inclination. To analyze the reason, when the specimen containing interface inclination is subjected to vertical load, the interface inclination changes the direction of vertical load transfer, part of which is transformed into normal stress perpendicular to the inclined plane, and the other part is transformed into downward shear force parallel to the inclined plane, at which time, the interface shear force causes the interface to produce slip damage. Therefore, the interface inclination angle has a large deterioration effect on the compressive strength of the sandstone-concrete binary. With the increase in the number of freeze–thaw cycles, the depression of the compressive phase of the stress–strain curve of the specimen is more obvious, indicating that the pores of the specimen are more easily compressed by the freeze–thaw cycles.

As shown in Figs. 7b–d and 8, the *UCS* of the sandstone-concrete composite specimens under the action of freeze–thaw cycles is basically consistent with the variation pattern of *NMR* porosity, and the increase of the number of freeze–thaw cycles has a deterioration effect on the strength of the specimens. The uniaxial strength decreases linearly with the increase in freeze–thaw cycles. The *UCS* decreases linearly with the increase of freeze–thaw cycles. The *UCS* of sand-concrete composite specimens with 0°, 45° and 90° interface inclination angles decreases to 29.72 MPa, 20.86 MPa and 23.14 MPa, respectively, which decreases by 44.98%, 45.39%, and 43.26% compared with the compressive strength after 0 freeze–thaw cycles. During the whole freeze–thaw cycle, the first 20 cycles led to the most obvious deterioration of compressive strength, and the strength loss rate of the composite specimens with 0°, 45°, and 90° bonding interface inclination reached 17.07%, 16.92%, and 14.49%, respectively. The 20–40 freeze–thaw cycles resulted in a slower strength loss rate, and the 40–60 freeze–thaw cycles resulted in an increased strength loss rate in the ring, but the increase was smaller than that of the first 20 freeze–thaw cycles.

### Correlation analysis between microscopic pore structure and macroscopic mechanical strength

**Figure 9:**
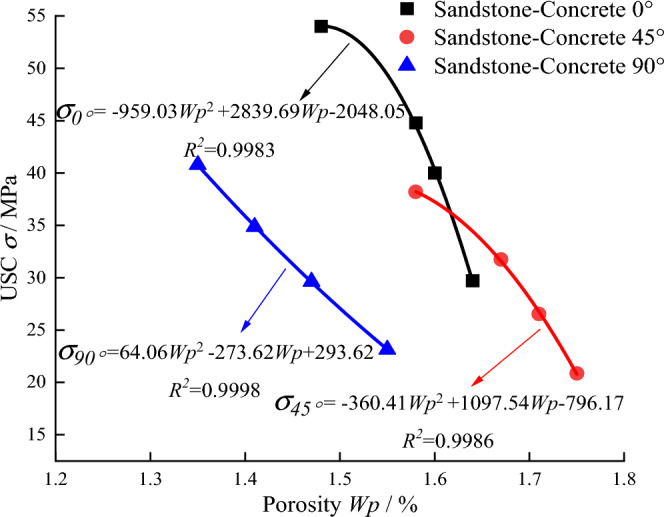
Relation between microstructure and macroscopic strength of sandstone-concrete composite specimens.

The relationship between microscopic porosity and macroscopic *UCS* of sandstone-concrete composite specimens with different cementation interface inclination angles and the fitted curves are shown in Fig. 9. Figure 9 shows that the *UCS* of sandstone-concrete composite specimens with different cementation interface inclination angles decreases parabolically with increased porosity. The variation is consistent with the change of mechanical indexes of sandstone and concrete during freeze–thaw cycles.

**Figure 10:**
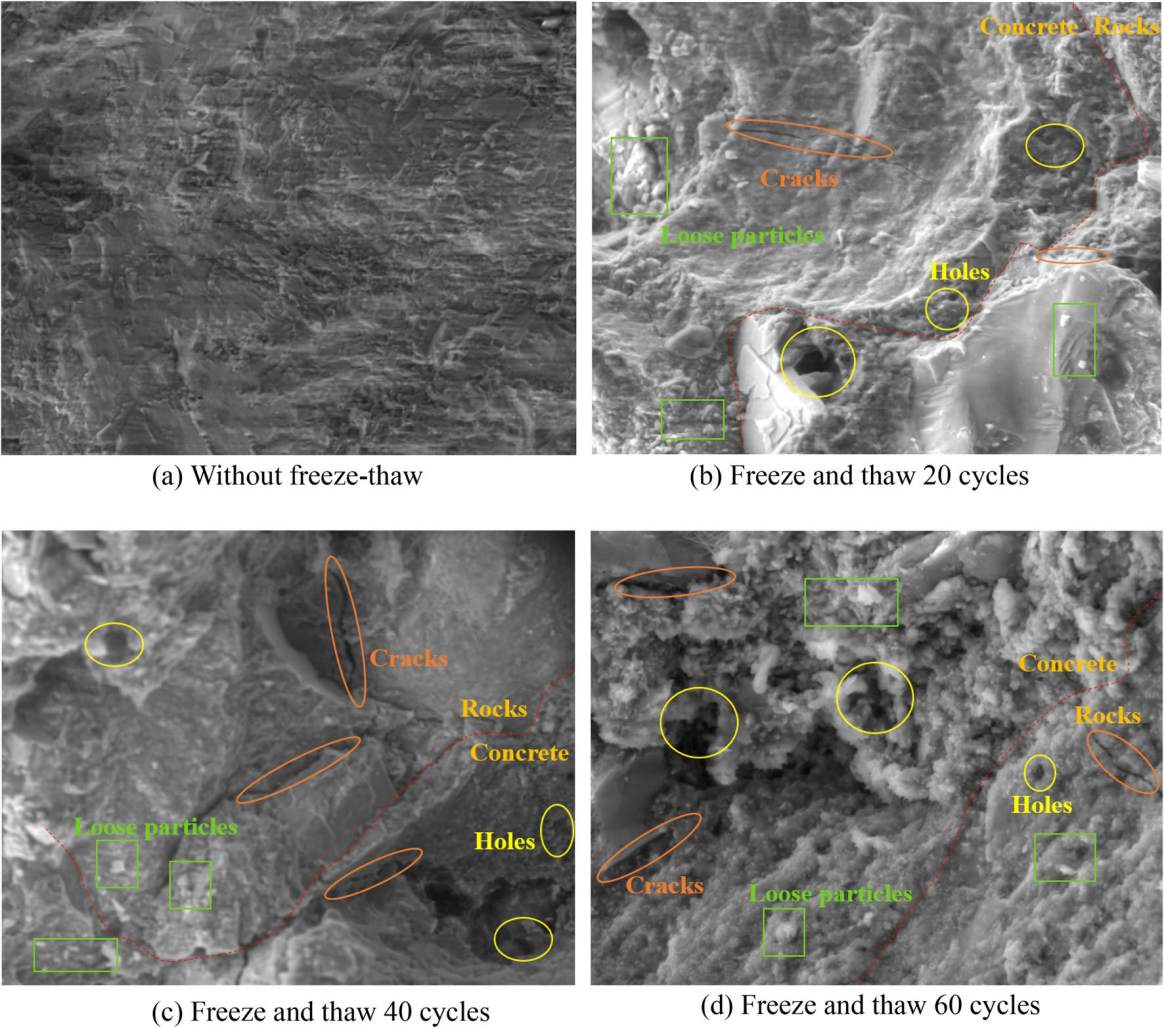
*SEM* images of specimens at different freeze–thaw cycles.

Figure 10 shows the electron microscope scans of the sandstone-concrete composite specimens without freeze–thaw and with 60 freeze–thaw cycles at 800 × pixels. Figure 10a shows that the unfreeze-thawed sandstone-concrete composite specimen has a dense structure with a few pores on the bonding surface, compact arrangement between the particles, complete cementation surface, flat surface and less attached small particles. After 20 cycles of freeze-thawing, the structure of the particles became loose, accompanied by a small number of small holes and micro-cracks, but the development depth of the pores and cracks was not obvious, as shown in Fig. 10b. After the 40th freeze-thawing, the number of cracks and holes in the specimens increased, and the holes gradually became larger, and the particles were more clearly shown in Fig. 10c. After the 60th freeze-thawing, the number of holes and cracks increased significantly compared with the previous freeze–thaw cycles and the depth of holes and cracks became deeper. The surface became uneven, and there were significantly large particles with clear particle boundaries. The adhesion between the particles was greatly weakened, and the structure became loose, as shown in Fig. 10d.

The above analysis indicates that the change of pore structure of sandstone-concrete binary, i.e., microscopic damage, is closely related to the number of freeze–thaw cycles. The concrete has relatively few pores, so saturated free water entering the pores and condensing into ice has little effect on the overall pore structure of the sandstone-concrete binary. Whereas the sandstone itself is rich in micro-fractures and micro-porosity, and its structure is loose, so free water is more likely to infiltrate, making the sandstone gather a large amount of pore water inside, which plays a dominant role in the microstructural damage of the sandstone-concrete binary under freeze–thaw conditions. Concrete, although less strong than sandstone, is prone to cracking when subjected to expansion force, and theoretically, porosity development should be more pronounced. However, the total pore volume of sandstone is larger than that of concrete. The relative content of large pores is high, and the good water stability of concrete is not easy to react with water. In contrast, the free water enters the sandstone interior to soften the sandstone mineral particles, which promotes the development and expansion of pore space and connectivity. Therefore, in the process of a continuous freeze–thaw cycle, the sandstone has a more pronounced accumulated damage than concrete and a higher sensitivity to freeze–thaw temperature difference than concrete. Due to the different sensitivity of sandstone and concrete to freeze–thaw temperature difference, when in the freeze–thaw environment, the expansion force at the cemented interface is of different magnitudes, and to achieve internal equilibrium, the sandstone-concrete material shows uncoordinated deformation. As a result, the freeze–thaw cycle further reduces the bond strength of the cemented interface and forms a structurally weak surface.

**Figure 11:**
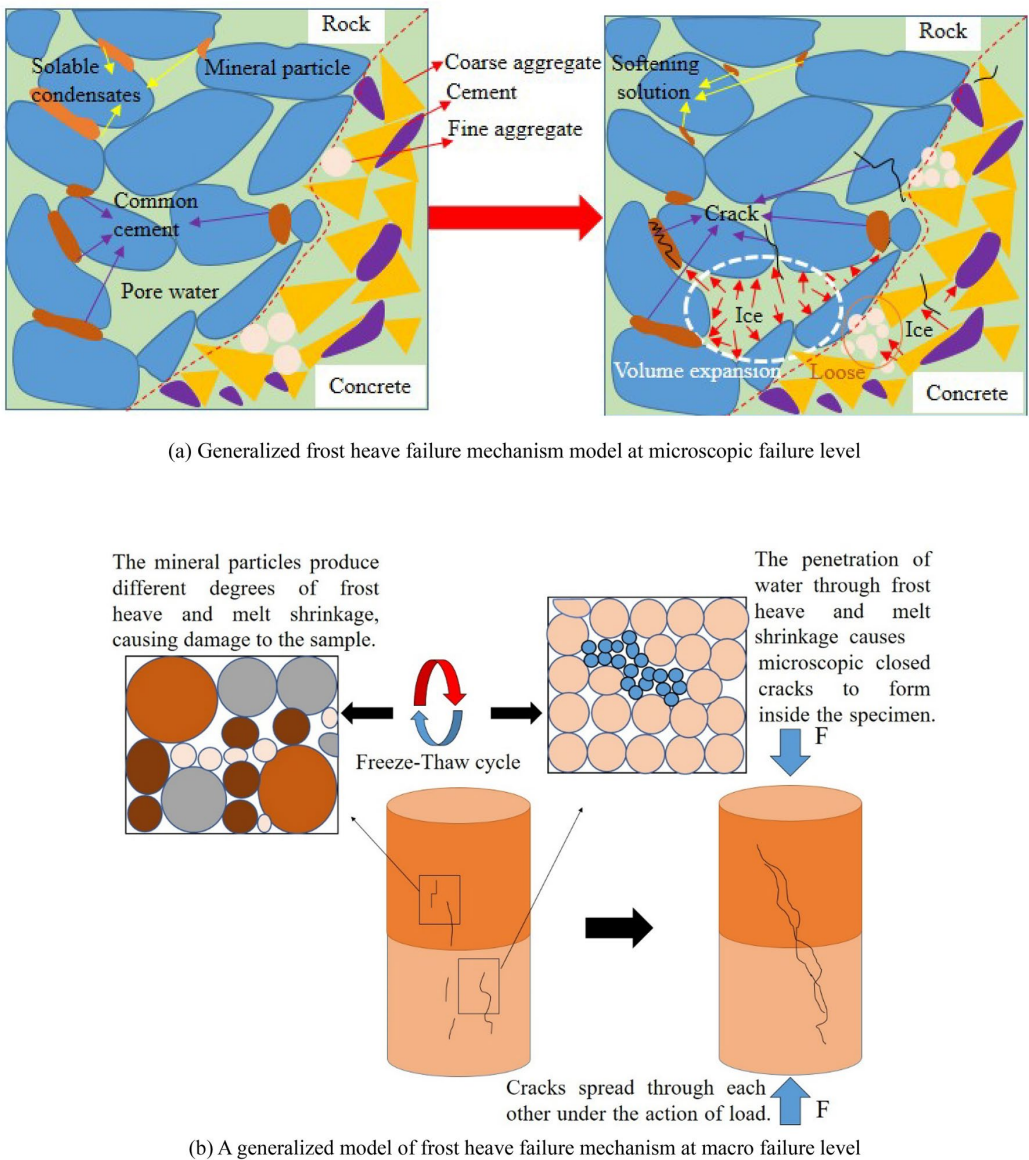
Generalized model of frost heave failure mechanism of sandstone-concrete binary.

The mechanical properties of the composite sandstone-concrete specimens under a freeze–thaw environment are developed comprehensively, and a generalized model of the microscopic frost damage mechanism of the sandstone-concrete binary shown in Fig. 11 is proposed.

As can be seen from Fig. 11, during the freeze–thaw process, the pore water freezes and dissolves reciprocally, and the dissolution of soluble mineral particles and fine aggregate inside the specimen weakens the cementation strength and cohesion. Furthermore, the pore water freezes into ice to expand the initial micro-pore expansion, forming an extrusion effect on the surrounding mineral particles. When the expansion force generated by water freezing is greater than the cementation force between the particles or the strength of the particles themselves, it will occur along the weak side of the structure of freezing and rupture, and the strength is significantly reduced. With the increase in the number of cycles, rupture cracks are also more and more developed from small pores to medium pores and from medium to large pores. The more pore water into the interior of the rock, the stronger the softening effect is, the more intensified the deterioration degree. Therefore, the porosity of the sandstone-concrete composite specimen gradually increases, and the compressive strength decreases in the freeze–thaw process.

## Conclusion

1. The NMR *T*_2_ spectra of concrete had two spectral peaks, while sandstone and sandstone-concrete binary had three spectral peaks, and the signal intensity amplitude of sandstone-concrete composite specimens was between the two materials of sandstone and concrete. The freeze–thaw cycles did not change the morphology of the *T*_2_ spectral curves of various specimens but promoted the pore development of various specimens.
2. The *T*_2_ spectral area of all types of specimens tended to increase with more freeze–thaw cycles. After 60 freeze–thaw cycles, the *T*_2_ spectral area of sandstone specimens increased by 28.92%, while that of concrete specimens increased by only 8.71%. The results indicate that the rock is more sensitive to temperature freeze–thaw cycles than concrete. In the process of 0–20 freeze–thaw cycles, *T*_2_ spectral area growth rate is the largest, 40–60 cycles next, and 20–40 cycles in the process of the smallest, indicating that the initial low temperature of freeze–thaw can cause obvious irreversible damage to the specimens, with freeze–thaw continued damage will also continue to occur, and when the number of cycles exceeds a certain threshold, the specimen porosity development speeds up and the irreversible damage intensifies.
3. The *UCS* of sandstone-concrete composite specimens is between the strength of sandstone and concrete and is affected by the dip angle of the cementation interface, which varied in a *V*-shape with the increase of the dip angle. During the freeze–thaw cycles, the change of compressive strength of sandstone-concrete composite specimens with the number of freeze–thaw cycles is basically consistent with the deterioration of microscopic porosity with the number of freeze–thaw cycles. The compressive strength deterioration was most obvious in the first 20 freeze–thaw cycles, and the rate of strength loss slowed down in 40–60 cycles, and the ring increase of strength loss rate in 40–60 cycles was smaller than that in the first 20 cycles.

## Data Availability

All the data in the tests of this study have been listed in the paper.
